# Unique spatiotemporal requirements for intraflagellar transport genes during forebrain development

**DOI:** 10.1371/journal.pone.0173258

**Published:** 2017-03-14

**Authors:** John Snedeker, Elizabeth N. Schock, Jamie N. Struve, Ching-Fang Chang, Megan Cionni, Pamela V. Tran, Samantha A. Brugmann, Rolf W. Stottmann

**Affiliations:** 1 Division of Human Genetics, Department of Pediatrics, Cincinnati Children’s Hospital Medical Center, Cincinnati, Ohio, United States of America; 2 Division of Developmental Biology, Department of Pediatrics, Cincinnati Children’s Hospital Medical Center, Cincinnati, Ohio, United States of America; 3 Division of Plastic Surgery, Department of Surgery, Cincinnati Children’s Hospital Medical Center, Cincinnati, Ohio, United States of America; 4 Department of Anatomy and Cell Biology, University of Kansas Medical Center, Kansas City, Kansas, United States of America; Justus Liebig Universitat Giessen, GERMANY

## Abstract

Primary cilia are organelles extended from virtually all cells and are required for the proper regulation of a number of canonical developmental pathways. The role in cortical development of proteins important for ciliary form and function is a relatively understudied area. Here we have taken a genetic approach to define the role in forebrain development of three intraflagellar transport proteins known to be important for primary cilia function. We have genetically ablated *Kif3a*, *Ift88*, and *Ttc21b* in a series of specific spatiotemporal domains. The resulting phenotypes allow us to draw several conclusions. First, we conclude that the *Ttc21b* cortical phenotype is not due to the activity of *Ttc21b* within the brain itself. Secondly, some of the most striking phenotypes are from ablations in the neural crest cells and the adjacent surface ectoderm indicating that cilia transduce critical tissue—tissue interactions in the developing embryonic head. Finally, we note striking differences in phenotypes from ablations only one embryonic day apart, indicating very discrete spatiotemporal requirements for these three genes in cortical development.

## Introduction

Primary cilia are microtubule based extensions of the plasma membrane with distinct proteomes, membrane composition and signaling dynamics. Microtubules nucleate from centrosomes at the base of the growing cilium and a number of proteins regulate transport, both away from the cell body towards the distal tip (anterograde) and back (retrograde). Based on transport paradigms in other organisms, these are collectively referred to as intraflagellar transport (IFT) proteins and complex-B proteins regulate the anterograde transport, while IFT-A complexes regulate retrograde transport. The primary cilium is an organelle that has undergone a renaissance in the field of developmental biology. This renewed interest has been due to an established role of the cilium for the proper regulation of a number of important developmental pathways. These pathways linked to the cilium include Wnt, PDGF, and IGF [1]. However, the most tantalizing connections between the primary cilium and a pathway have been drawn with the Sonic hedgehog (Shh) signaling pathway. Current models suggest that cilia exert influence on signaling pathways through a combination of differential receptor localization and/or transcription factor processing, especially Gli proteins. Some of the initial data came from forward genetic studies where unbiased mutagenesis screens have identified several genes important for primary cilia form and function to regulate Shh signaling [2, 3].

Mutations in ciliary genes cause a class of diseases referred to as ciliopathies which affect many different organ systems and have been shown to frequently present with intellectual disability [4, 5]. More profound central nervous system defects have been seen in animal models of ciliopathies but these are often associated with other syndromic conditions which are lethal in human [6].

Three genes known to be important for primary cilium form and function are *tetratricopeptide repeat domain 21b (Ttc21b)*, *kinesin family member 3a* (*Kif3a*) and *intraflagellar transport 88* (*Ift88*). *Ttc21b* (also known as *Thm1* and *Ift139*) was originally identified in a mouse forward genetic screen for late stage organogenesis defects where the *alien* allele had pleiotropic effects [7]. The *Ttc21b*^*aln/aln*^ mutants are perinatal lethal and show increased Shh signaling in multiple tissues, presumably as a result of abnormal processing of Gli proteins which are known to be important for Shh regulation [8]. Further studies showed that abnormal Hedgehog signaling in these mutants contributes to mispatterning of the embryonic forebrain [6] and to polycystic kidneys [9]. The cellular basis for these defects became clearer upon cloning of the mutated gene as *Ttc21b* which is part if the IFT-A complex required for proper rates of retrograde transport of cargo from the distal tip of the cilium back into the body of the cell (see [10] for a full review of retrograde IFT). Consistent with this model, studies of ciliary trafficking in the *Ttc21b*^*aln/aln*^ mutants demonstrated a reduction in the rate of retrograde IFT [8]. *Kif3a* was initially identified as an axonal transport molecule [11] and a null mouse allele revealed a role for ciliogenesis in the embryonic node [12]. *Kif3a* is one subunit of the heterotrimeric kinesin complex which is responsible for the anterograde transport of the IFT trains along the axoneme from the centrosome to the distal tip of the cilium [13, 14]. In most contexts, mutations in *Kif3a* are associated with loss or reduction of Shh signaling [3], but in the craniofacial tissues loss of *Kif3a* leads to an increase in Shh signaling [15]. *Ift88* was initially identified in a mouse model of polycystic kidney disease [16, 17] and is also required for proper Shh signaling [3]. IFT88 is a protein within the approximately ten-member “IFT-B core” which forms a large complex linking cargo to the anterograde kinesin motor for trafficking to the distal tip of the cilium (for review, see [18]). Thus, these three genes together represent different, but related, aspect of trafficking within the cilium necessary for proper cilia form and function, and consequently, embryogenesis. Craniofacial and/or CNS defects have been demonstrated from loss of function of *Kif3a* [15], *Ift88*, and *Ttc21b* (our own unpublished observations).

Increasing evidence suggests that not all tissues interpret the loss of primary cilia in the same manner. To begin to refine our understanding of how cilia regulate forebrain development, we utilized conditional alleles of *Ttc21b*, *Kif3a* and *Ift88* in combination with Cre transgenic alleles to ablate these genes in the presumptive forebrain, the definitive forebrain, the neural crest cells surround the forebrain and in the surface ectoderm. Together this series of ablations has revealed a series of striking spatiotemporal requirements for these genes in development of the forebrain. In combination with similarly dynamic requirements in craniofacial development (Schock et al., this issue), we propose different tissues utilize cilia to modulate developmental signaling in tissue-specific and stage-specific contexts.

## Materials and methods

### Mouse strains and husbandry

All mouse alleles used in this study have been previously published: *Ttc21b*^*aln*^ is an ENU-induced null allele [8]; *Ttc21b*^*tm1a(KOMP)Wtsi-lacZ*^ was used for lacZ expression and was mated with a germline FLP recombinase line to remove the gene trap to create a conditional *Ttc21b*^*tm1c(KOMP)Wtsi-lacZ*^ (*Ttc21b*^*flox*^) allele [9]; *Kif3a*^*tm2Gsn*^ (*Kif3a*^*flox*^)[19]; *Ift88*^*tm1Bky*^ (*Ift88* ^*flox*^*)*[20]; FVB/N-Tg*(EIIa-cre)C5379*^*Lmgd*^/J (*EIIa-Cre*) [21]; B6.129S2-*Emx1*^*tm1(cre)Krj*^/J (*Emx1-Cre*)[22]; *129(Cg)-Foxg1*^*tm1(cre)Skm*^/J *(Foxg1-Cre)*[23]; *B6*.*Cg-Tg(Wnt1-cre)*^*11Rth*^(*Wnt1-Cre*) [24]; *Tfap2a*^*tm1(cre)Moon*^ (*Ap2a-Cre*) [25]; *B6;129S4-Gt(Rosa)26Sor*^*tm1Sor*^*/J* (*R26R)*[26]; and B6.129(Cg)-*Gt(ROSA)26Sor*^*tm4(ACTB-tdTomato*,*-EGFP)Luo*^*/J* (*ROSA*^*dTom/EGFP*^)[27]. Published protocols were used for all genotyping except for the *Ttc21b*^*aln*^ allele where a custom Taqman assay was employed (Invitrogen; details available upon request). Timed matings were established and noon on the day of mating plug was designated embryonic day (E) 0.5. This study was carried out in strict accordance with the recommendations in the Guide for the Care and Use of Laboratory Animals of the National Institutes of Health. Animals were housed at or below IACUC determined densities with AALAC-approved veterinary care and fed Autoclavable Mouse Breeder Diet 5021 (LabDiet, St. Louis, MO). The protocol was approved by the Institutional Animal Care and Use Committee of the Cincinnati Children’s Hospital Medical Center (protocol numbers 1D05052 and IACUC2013-0068). All euthanasia (cervical dislocation followed by thoracotomy) and embryo harvests were performed after isoflurane sedation to minimize animal suffering and discomfort.

### Embryo collection & microscopy

Embryos were harvested via Caesarian section and dissected, examined and photographed with a Zeiss Discovery.V8, Axiocam MRc5 and AxioVision software. Brain measurements were done within the Axiovision software suite and tabulated with Excel. Student t-tests were performed to measure significance of forebrain measurements.

### Histology & immunohistochemistry

Embryos analysis were fixed with formalin for at least twenty-four hours and processed for paraffin embedding. Sections were cut at a thickness of 14μm and stained with hematoxylin and eosin using standard techniques. The TuJI antibody (SIGMA) was used at 1:500 for 2 hours at room temperature on paraffin sections with citrate buffer antigen retrieval with standard DAB staining. Embryos were stained for lacZ using standard protocols [28].

## Results

### Multiple Cre recombinase transgenics used to genetically ablate cilia genes

**Fig 1:**
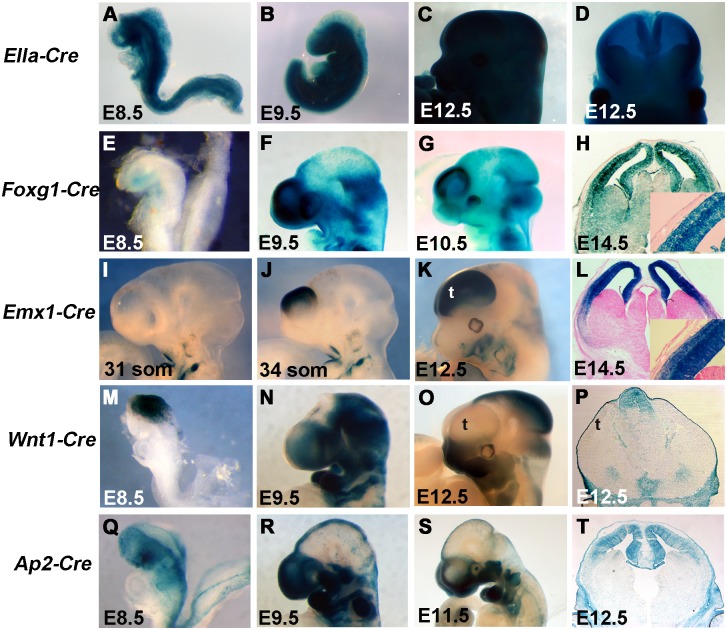
Recombination pattern of Cre transgenics used to ablate cilia genes. The pattern of Cre-mediated recombination with the *R26R-lacZ* reporter is shown for all lines used. (A-D) *EIIa-Cre* is expressed at high levels throughout the embryo with some mosaicism, including the entire nervous system at early organogenesis stages (D). (E-H) *Foxg1-Cre* is highly expressed in the developing telencephalon from the earliest stages of formation but not in the overlying surface ectoderm (H). (I-L) *Emx1-Cre* is specific to the dorsal telencephalon with recombination evident between 31 and 34 somite (~E10.5). Note the later onset and more specific recombination as compared to *Foxg1-Cre*. (M-N) *Wnt1-Cre* activity is seen in the midbrain and dorsal midline of the neural tube and in the emerging neural crest cells populating the craniofacial tissues (N,O). Note expression is not seen in the telencephalon (P). *Ap2-Cre* recombination is detected in the dorsal midline and neural crest like *Wnt1-Cre*, but also in the dorsal telencephalon (T). (t = telencephalon)

We used a series of Cre-Recombinase expressing mouse transgenic alleles to genetically ablate primary cilia genes in a number of complementary expression patterns [21–25]. The FVB/N-Tg*(EIIa-cre)C5379*^*Lmgd*^/J transgene (called *EIIa-Cre* here) expresses Cre under the control of the adenovirus EIIa promoter [21]. Expression is somewhat mosaic but begins in the very early embryo and can be used to delete a gene of interest through all or most of the embryo to recapitulate germline null allele phenotypes. Consistent with this expression pattern, we generated *EIIa-Cre; R26R* embryos in which the pattern of Cre recombination is marked by ß-galactosidase expression from the Cre reporter *B6;129S4-Gt(Rosa)26Sor*^*tm1Sor*^*/J* (*R26R*) and is seen through the majority of the embryonic tissue we analyzed between embryonic day (E) E8.5—E12.5 (Fig 1A–1D). In order to ablate genes specifically in the developing forebrain, we utilized the *129(Cg)-Foxg1*^*tm1(cre)Skm*^/J *(Foxg1-Cre)* and *B6*.*129S2-Emx1*^*tm1(cre)Krj*^*/J* (*Emx1-Cre*) mouse lines. *Foxg1-Cre* expresses Cre recombinase from the endogenous *Foxg1* locus [23] and is one of the earliest known acting Cre’s in the developing mouse forebrain. Consistent with the literature [23], we first noted Cre recombination activity in the developing foregut region at E8.5 (Fig 1E). We observed expression in the early anterior neural ridge and telencephalic vesicle during E8 and strong recombination activity in the telencephalon by E9.5 (Fig 1F) and continuing through E14.5 (Fig 1G and 1H). Consistent with the known recombination pattern of *Foxg1-Cre* on some genetic backgrounds [23], we did see recombination extending beyond the developing telencephalon in a proportion of our embryos (Fig 1E–1H) but the Cre activity was clearly highly active in the dorsal forebrain. As in previous studies [22], the *Emx1-Cre* transgenic is active slightly later in cortical development. *Emx1-Cre;R26R* embryos did not show Cre recombination activity in the telencephalon at E10.25 (Fig 1I) but did indicate robust recombination by E10.5 (Fig 1J). We detected recombination activity throughout the pallium at E12.5 and E14.5 (Fig 1K and 1L). In combination, the *Foxg1-Cre* and *Emx1-Cre* allow genetic ablation of “floxed” genes in the forebrain at different stages with the *Foxg1-Cre* active approximately 36 hours earlier than *Emx1-Cre*. Consistent with the expression of *Foxg1* and *Emx1*, we see very little or no Cre recombinase activity in the surface ectoderm (see insets in Fig 1H and 1L). In order to address the caveats associated with *Foxg1-Cre* ectopic recombination, we have incorporated a Cre reporter into our experimental design as described below to identify embryos with desired patterns of Cre activity.

We also hypothesize the primary cilia genes we are interested in may affect brain development from non-neural sources such as the neural crest cells and/or surface ectoderm surrounding the developing brain. In order to address these hypotheses, we used the *Wnt1-Cre* (*B6*.*Cg-Tg(Wnt1-cre)*^*11Rth*^; [24]), and *Ap2-Cre* (*Tfap2a*^*tm1(cre)Moon*^; [25]) alleles. The *Wnt1-Cre* is a transgenic expressing Cre via the *Wnt1* enhancer [24, 25] and has often been shown to act in very early neural crest cells as they are generated at the dorsal midline in the endogenous *Wnt1* expression domain [24, 29–32](Fig 1M). Cre reporter activity after recombination was then continuously detected in the midbrain and NCCs as they migrate from the *Wnt1* domain to populate the developing craniofacial tissues (Fig 1N and 1O). *Wnt1-Cre* activity was not noted in the neural tissue of the developing forebrain but we did note expression in the tissue around the developing forebrain at E12.5 (Fig 1P), consistent with the known lineage of NCC’s contributing to the frontal bone and meninges overlying the telencephalon [30]. The AP2-Cre allele is designed as an IRES-Cre insertion into the *transcription factor AP-2*, *alpha* (*Tfap2a*, formerly *Ap2)* genomic locus [25]. Regions of *Tfap2a* expression include the pharyngeal NCC’s and ectoderm. Consistent with this transgenic design, Cre activity was detected as early as E8.5 in the ectoderm (Fig 1Q) and continues to look quite similar to *Wnt1-Cre* thereafter (Fig 1R, 1S and 1T) with the added neural domain (Fig 1T). The critical difference for our studies is the broader expression of *Ap2-Cre* as compared to *Wnt1-Cre* in the early anterior embryo (cf. Fig 1M and 1Q).

### Forebrain ablation of *Ttc21b* does not recapitulate the *Ttc21b*^*aln*^ phenotype

**Fig 2:**
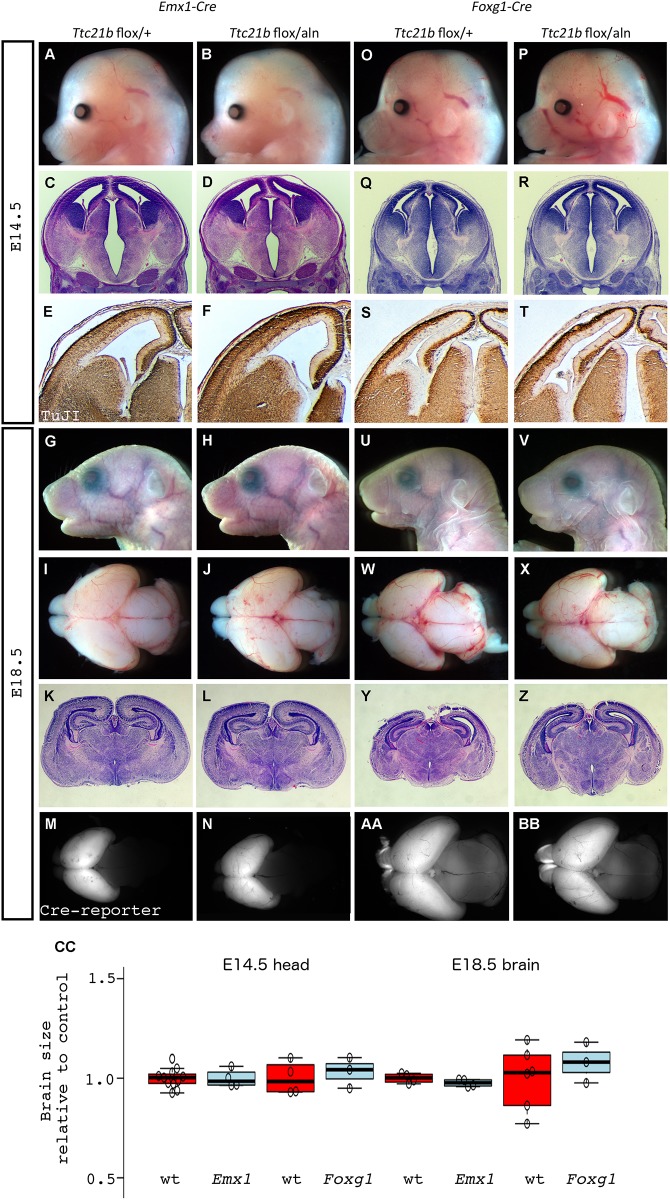
Deletion of *Ttc21b* from solely the developing forebrain does not lead to cortical malformation. (A-N) *Emx1-Cre* was used to delete a conditional allele of *Ttc21b* but does not lead to morphological (A,B,G,H,I,J), histological (C,D,K,L) or neural differentiation (immunohistochemistry for TuJI in E,F) phenotypes. Control and *Emx1-Cre; Ttc21b*^*flox/aln*^ embryos are shown at E14.5 (A-F) and E18.5 (G-N).. *Foxg1-Cre* deletions also do not cause cortical phenotypes at E14.5 (O-T) or E18.5 (U-Z). Cre recombination patterns for each genotype are shown with the *ROSA*^*dTom/EGFP*^ reporter allele (M,N,AA,BB). All paired images are at the same magnification. (t = telencephalon) (CC) Quantification for brain sizes are normalized to control for each respective experiment. Center lines show the medians; box limits indicate the 25th and 75th percentiles as determined by R software; whiskers extend 1.5 times the interquartile range from the 25th and 75th percentiles, outliers are represented by dots; data points are plotted as open circles. n = 11, 4, 4, 3, 4, 4, 6, 3, respectively. Grey = wt, Red = mut

We previously noted that loss of function of *Ttc21b* in the homozygous *Ttc21b*^*aln/aln*^ mutant embryos led to profound forebrain defects [6, 8]. Among these were a dramatic reduction in size of the telencephalon and a loss of anterior neural character in favor of an expansion of midbrain fate. We also noted a disorganization of the neuroepithelium within the *Ttc21b*^*aln/aln*^ cortex. In order to further understand the molecular mechanisms underlying this phenotype, we first sought to use a conditional allele of *Ttc21b* [9] to ablate *Ttc21b* in the early telencephalon using *Emx1-Cre* with the intent of studying the role of *Ttc21b* in the developing forebrain independent of the earlier embryonic patterning phenotype. Intriguingly, the forebrain microcephaly phenotype seen in the *Ttc21b*^*aln/aln*^ mouse was not recapitulated in the *Emx1-Cre;Ttc21b*^*flox/aln*^ embryos (Fig 2A–2N). In fact, we did not see any obvious morphological differences between control and mutant brain morphology at either E14.5 (Fig 2A–2D) or E18.5 (Fig 2G–2L), either in whole mount or histological analyses. For all of our genotypic classes we quantified head size at E14.5 and brain size at E18.5. Neither of these were affected in the *Emx1-Cre;Ttc21b*^*flox/aln*^ embryos with mutant head size being 99.8% of control at E14.5 and 97.6% at E18.5 (Fig 2CC; p = 0.95 and 0.62, respectively). We also performed immunohistochemistry for TuJ1 to highlight differentiated neurons and again saw no difference between mutant and control embryos (Fig 2E and 2F). We additionally observed that *Emx1-Cre;Ttc21b*^*flox/aln*^ mutants were capable of surviving into adulthood with no overt behavioral phenotypes or increased morbidity as compared to controls (data not shown). To verify that the Cre recombinase activity occurred as expected, we also incorporated a dual reporter of Cre activity in these crosses. The Cre reporter allele (B6.129(Cg)-*Gt(ROSA)26Sor*^*tm4(ACTB-tdTomato*,*-EGFP)Luo*^*/J*; hereafter *ROSA*^*dTom/EGFP*^) will produce dTomato protein prior to Cre activity, and EGFP after. As expected, the *Emx1-Cre* crosses produce embryos with EGFP signal in the forebrain (Fig 2M and 2N) and dTomato signal everywhere else (data not shown).

We reasoned that the *Emx1-Cre* ablation at E10.5 (Fig 1J) might occur too late in development to recapitulate the embryonic microcephaly of the *Ttc21b* null allele and took advantage of the earlier Cre activity in the *Foxg1-Cre* mouse (Fig 1E–1G). At E14.5, we observed no difference in E14.5 *Foxg1-Cre;Ttc21b*^*flox/aln*^ embryos as compared to control (Fig 2O–2R, CC; 103% of control brain size, p = 0.16). We did note subtle alterations in the pattern of differentiated neurons with TuJ1 immunohistochemistry (Fig 2S and 2T). Similarly, we noted no obvious differences between *Foxg1-Cre;Ttc21b*^*flox/aln*^ embryos and control at E18.5, either in whole mount (Fig 2U–2X; 107% relative brain size, p = .45), or upon histological analysis (Fig 2Y and 2Z). Again, we used the Cre reporter allele to show that the recombination was occurring in the forebrain as expected (Fig 2AA and 2BB). We conclude from these data that ablation of *Ttc21b* in the forebrain is, surprisingly, insufficient to recapitulate the microcephaly we observed in the *Ttc21b*^*aln/aln*^ null embryos.

### Germline ablation of *Ttc21b* does recapitulate the *Ttc21b*^*aln*^ phenotype

**Fig 3:**
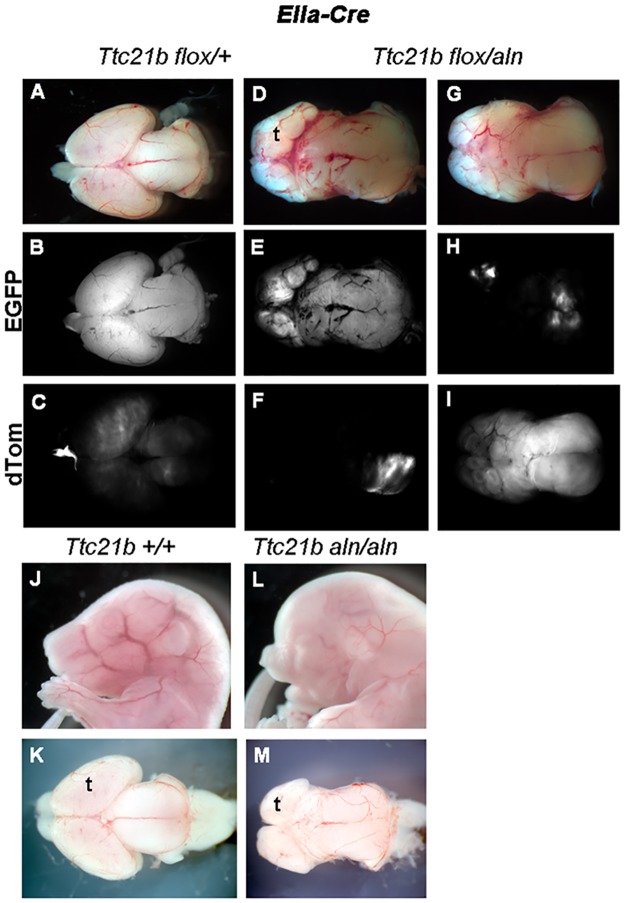
Deletion of *Ttc21b* with *EIIa-Cre* phenocopies homozygous *Ttc21b*^*aln/aln*^ embryos. Genetic ablation of *Ttc21b* with the EIIa-Cre creates microcephalic brains (D,G) which are similar to that seen in homozygous null embryos (L,M). The *ROSA*^*dTom/EGFP*^ reporter allele shows the mosaic nature of some *EIIa-Cre* embryos where EGFP expression (B,E,H) marks recombined tissue and dTom expression (C,F,I) indicates tissue without Cre activity. All paired images are at the same magnification. (t = telencephalon)

Given the surprising results in the *Emx1-Cre;Ttc21b*^*flox/aln*^ and *Foxg1-Cre;Ttc21b*^*flox/aln*^embryos, we performed a genetic ablation throughout the embryo to ensure the fidelity of the *Ttc21b* conditional allele. We used the *EIIa-Cre* allele to create *EIIa-Cre;Ttc21b*^*flox/aln*^ embryos. Both the overall appearance and brain morphology of these mutants were similar to the *Ttc21b*^*aln/aln*^ phenotype (compare Fig 3D, 3G and 3M). We also noted the expression of *EIIa-Cre* can be mosaic and utilized the *ROSA*^*dTom/EGFP*^ reporter allele to precisely determine the patterns of Cre recombination in the mutant embryos. We recovered *EIIa-Cre;Ttc21b*^*flox/aln*^;* ROSA*^*dTom/EGFP*^ embryos which had GFP throughout the forebrain indicating virtually complete recombination (Fig 3E), but also embryos in which the GFP was expressed at relatively low levels in the microcephalic brain (Fig 3H). These findings are consistent with our previous data that Cre recombinase activity within the forebrain is not necessary to generate the microcephalic phenotype of *Ttc21b*^*aln/aln*^ null embryos. An alternative explanation for the normally sized forebrain in the *Emx1-Cre;Ttc21b*^*flox/aln*^ and *Foxg1-Cre;Ttc21b*^*flox/aln*^ embryos is a genetic background effect. We have noted a decreased severity of the microcephaly phenotype in *Ttc21b*^*aln/aln*^ mutants maintained on a C57BL/6J (B6) background as compared to FVB/NJ (FVB; our unpublished observations). To address this possibility, we independently crossed the *Ttc21b*^*flox*^, *Emx1-Cre* and *Foxg1-Cre* alleles at least two generations onto both the B6 and FVB backgrounds and generated *Emx1-Cre;Ttc21b*^*flox/aln*^ and *Foxg1-Cre;Ttc21b*^*flox/aln*^ from each backcross, respectively. No mutants from these crosses on any background have shown different phenotypes.

### *Ttc21b* expression is restricted during organogenesis in the mouse

**Fig 4:**
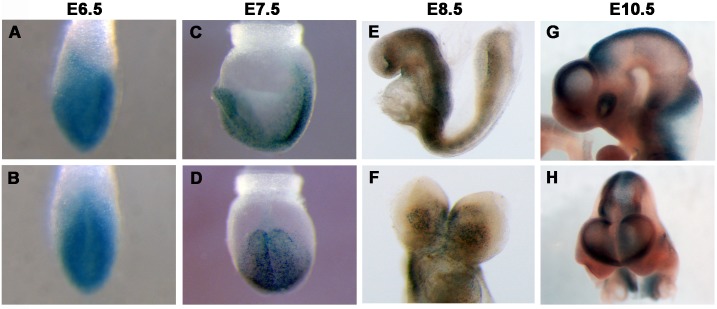
Expression of *Ttc21b*. *Ttc21b*^*lacZ*^ expression at E6.5-E10.5. Frontal views are shown in B,D,F,H.

In order to further explore the hypothesis that *Ttc21b* is required outside the developing forebrain to regulate brain size, we examined expression with the *Ttc21b*^*tm1a(KOMP)Wtsi-lacZ*^ conditional gene trap allele (*Ttc21b-lacZ*) [9]. At E6.5 and E7.5, *Ttc21b* is broadly expressed (Fig 4A–4D). E8.5 expression patterns are similar to our previous RNA *in situ* hybridization analysis [6] and show broad expression in the embryo with higher levels in the neural tube and somites but not particularly strong expression in the anterior neural tissues (Fig 4E and 4F). *Ttc21b* expression at E9.5 and E10.5 is again noted in multiple tissues affected in the *Ttc21b*^*aln/aln*^ mutants [7, 8] such as the limb, eye and dorsal neural tube (Fig 4G and 4H). The expression in the dorsal neural tube and craniofacial tissues as well as the craniofacial phenotypes previously noted in the *Ttc21b*^*aln/aln*^ mutants [7] suggested the neural crest to be a region requiring *Ttc21b* activity.

### *Ttc21b* is required in neural crest cells and surface ectoderm to regulate forebrain size

**Fig 5:**
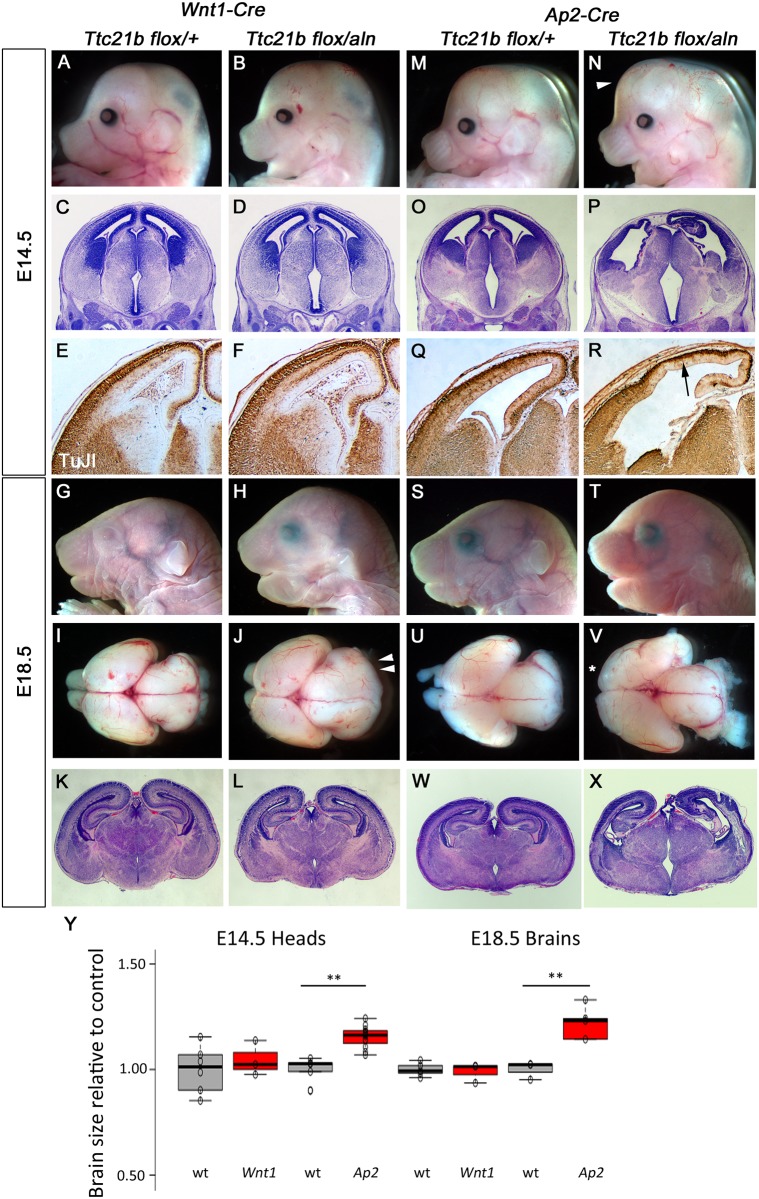
Deletion of *Ttc21b* from neural crest cells and surface ectoderm. (A-L) *Wnt1-Cre* mediated deletion of *Ttc21b* does not lead to morphological (A,B,G,H,I,J), histological (C,D,K,L) or neural differentiation (E,F) phenotypes in the forebrain at E14.5 (A-F) or E18.5 (G-L). The midbrain is enlarged at E18.5 (double arrowheads in J). (M-X) *Ap2-Cre; Ttc21b*^*flox/aln*^ embryos at E14.5 and E18.5 have an enlarged forebrain (arrowhead in N, V) with disrupted cortical architecture (P,X) and reduced numbers of differentiated neurons (R). Loss of olfactory bulbs is also noted at E18.5 (asterisk in V). All paired images are at the same magnification. (Y) Quantification for brain sizes. Center lines show the medians; box limits indicate the 25th and 75th percentiles as determined by R software; whiskers extend 1.5 times the interquartile range from the 25th and 75th percentiles, outliers are represented by dots; data points are plotted as open circles. n = 6, 3, 5, 12, 5, 3, 3, 5, respectively. (**: p <0.005).

In order to further explore the role of *Ttc21b* in anterior embryonic development and potentially determine the mechanism leading to the microcephaly in *Ttc21b*^*aln/aln*^ mutants, we used the *Wnt1-Cre* and *Ap2-Cre* alleles to ablate *Ttc21b* in NCC’s and both NCC’s and surface ectoderm, respectively (Figs 1 and 5). *Wnt1-Cre;Ttc21b*^*flox/aln*^ embryos showed no obvious morphological differences in brain development as compared to controls at E14.5 (Fig 5A and 5B, 104% of control, p = 0.55). Histological analysis and TuJI expression were also similar between mutant and control (Fig 5C–5F). However, *Wnt1-Cre;Ttc21b*^*flox/aln*^ embryos at E18.5 showed craniofacial phenotypes (Fig 5G and 5H; see Schock et al.,). Microdissection of the brain at this stage indicated a slightly enlarged midbrain in mutants as compared to controls (Fig 5I and 5J). However, histological analysis indicates that the enlargement does not result in any changes in the gross architecture of the mutant forebrain (Fig 5K and 5L; 98.9% of control, p = .708).

In contrast to the relatively mild phenotypes in the *Wnt1-Cre;Ttc21b*^*flox/aln*^ embryos, ablation of *Ttc21b* in both the NCCs and surface ectoderm (Fig 1Q–1T) in *Ap2-Cre;Ttc21b*^*flox/aln*^ embryos resulted in an obviously enlarged forebrain at E14.5 (Fig 5M and 5N; 115% of control, p = 6.5E-5). Histological analysis highlighted a disruption of normal tissue architecture (Fig 5O and 5P) and TuJI expression analysis showed a reduction in differentiated neurons (Fig 5Q and 5R). *Ap2-Cre;Ttc21b*^*flox/aln*^ embryos were also grossly abnormal at E18.5, although not as affected as might be predicted by the E14.5 phenotypes (Fig 5S and 5T). Micro-dissection of the E18.5 brain showed loss of olfactory bulbs, an 23% increase in forebrain size (p = 0.0050), and a grossly normal midbrain (Fig 5U and 5V). Ventriculomegaly of the lateral ventricles was detected along with variable dysmorphology of the hippocampus and cortical plate (Fig 5W and 5X).

### *Kif3a* has a role in forebrain development unique from *Ttc21b*

A null allele of *Ttc21b* revealed a role in retrograde trafficking and proper [8]. Our use of the conditional allele described here allowed much more specific conclusions about discrete spatiotemporal requirements for *Ttc21b* in normal CNS development. Two other genes important for ciliogenesis and anterograde transport within the cilium are *Kif3a* and *Ift88*. As conditional alleles exist for each of these, we took a similar approach to determine if there are also discrete spatiotemporal requirements for these primary cilia genes in CNS development.

**Fig 6:**
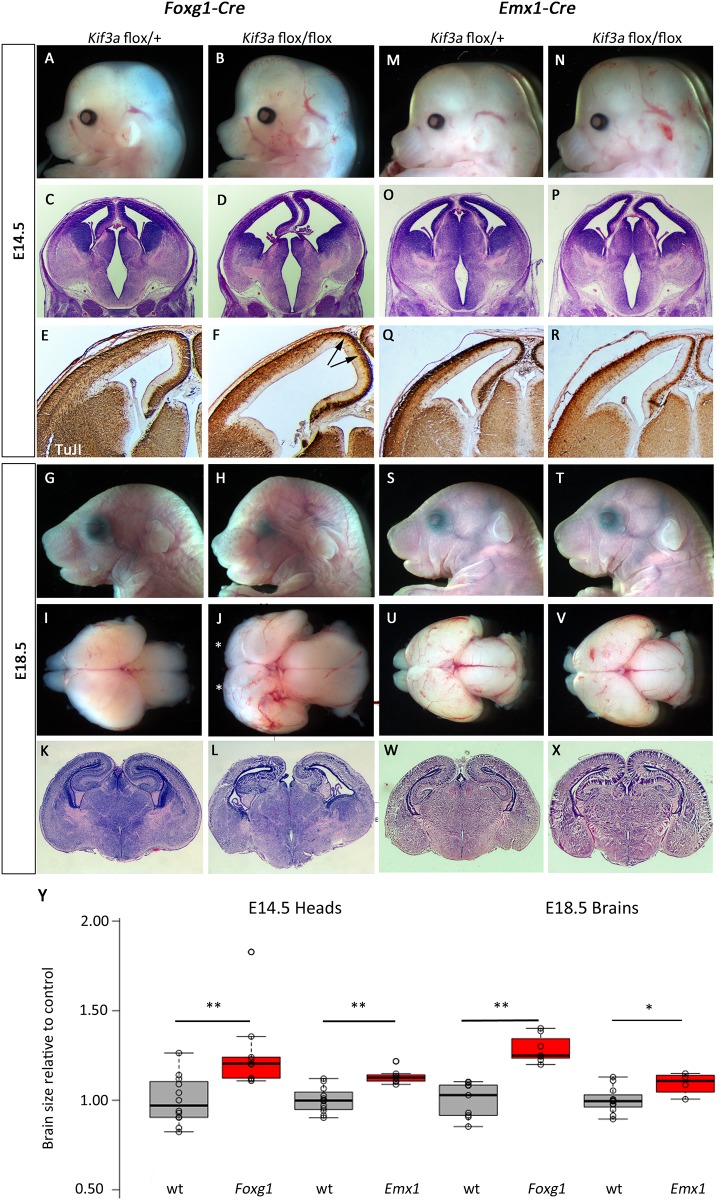
Deletion of *Kif3a* in early stages of forebrain development leads to increased brain size. (A-L) *Foxg1-Cre* was used to delete a conditional allele of *Kif3a* and the forebrain was enlarged at E14.5 (B) and E18.5 (H,J,). Fewer differentiated neurons are noted in medial regions at E14.5 (D,F, arrows show specific areas of decreased TuJI). Olfactory bulbs are absent at E18.5 in mutants (asterisks in J). (M-X) Similar enlargements are seen with *Emx1-Cre* ablation but the effects are much less severe. All paired images are at the same magnification. (Y) Quantification for brain sizes. Center lines show the medians; box limits indicate the 25th and 75th percentiles as determined by R software; whiskers extend 1.5 times the interquartile range from the 25th and 75th percentiles, outliers are represented by dots; data points are plotted as open circles. n = 12, 9, 12, 7, 9, 7, 12, 4, respectively. (*:p<0.05, **:p<0.005)

Early forebrain ablation (E9.5) of *Kif3a* using *Foxg1-Cre* led to overt developmental defects in the *Foxg1-Cre; Kif3a*^*flox/flox*^ mutant embryos. At E14.5, a slightly enlarged cranium was observed (Fig 6A and 6B; 126% size of control, p = 0.0043). Histological analysis revealed ventriculomegaly and a marked reduction in size of the ganglionic eminences (Fig 6C and 6D). TuJ1 expression at E14.5 showed a reduction in the number of differentiated neurons, especially in the extreme dorsal regions of the telencephalon (Fig 6E and 6F). At E18.5, *Foxg1-Cre; Kif3a*^*flox/flox*^ mutant embryos were notably dysmorphic with distinctive craniofacial features (Fig 6G and 6H) and microdissection of the brain revealed a significantly enlarged forebrain with loss of olfactory bulbs (Fig 6I and 6J; 128% of control, p = 2.1E-5). Histological analysis confirmed a generally dysmorphic telencephalon and ventriculomegaly (Fig 6K and 6L).

Surprisingly, ablation of *Kif3a* within the forebrain only slightly later (E10.5) using *Emx1-Cre* had a very different result. *Emx1-Cre; Kif3a*^*flox/flox*^ mutant embryos showed much more subtle enlargement of the forebrain at E14.5 (Fig 6M and 6N; 113% of control, p = 0.0005) and normal histology and differentiation (Fig 6O–6R; forebrain is 109% of control size, p = 0.045). This was also true at E18.5 (Fig 6S–6X). Furthermore, *Emx1-Cre;Kif3a*^*flox/flox*^ mice survive postnatally (data not shown).

**Fig 7:**
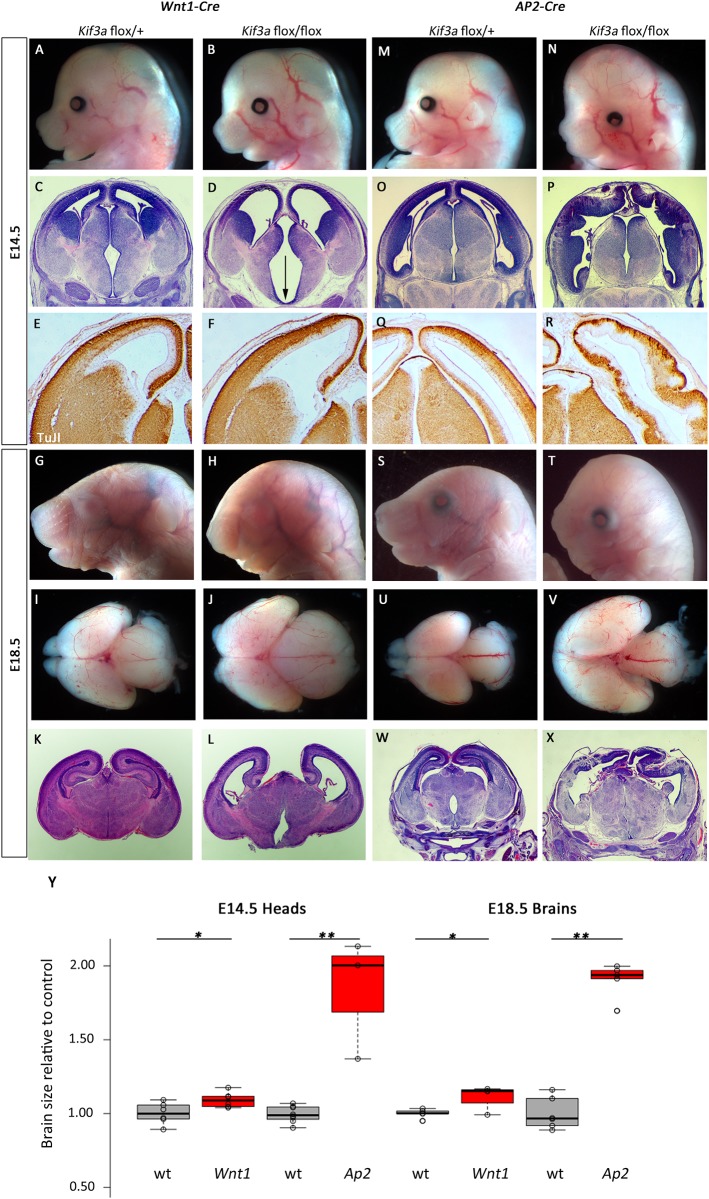
Deletion of *Kif3a* from neural crest and surface causes cortical malformation. (A-L) *Wnt1-Cre* mediated deletion of *Kif3a* causes morphological (A,B,G,H,I,J), and histological (C,D,K,L) phenotypes in the forebrain at E14.5 (A-F) and E18.5 (G-L). The third ventricle is enlarged at E14.5 (arrow indicates widened base of ventricle in D). (M-X) *Ap2-Cre; Kif3a*^*flox/flox*^ embryos at E14.5 and E18.5 have a profoundly enlarged forebrain (N, V) with disrupted cortical architecture (P,R,X). All paired images are at the same magnification. (Y) Quantification for brain sizes. Center lines show the medians; box limits indicate the 25th and 75th percentiles as determined by R software; whiskers extend 1.5 times the interquartile range from the 25th and 75th percentiles, outliers are represented by dots; data points are plotted as open circles. n = 6, 6, 8, 3, 5, 4, 6, 5, respectively. (*:p<0.05, **:p<0.0005)

Ablation of *Kif3a* in the neural crest and midbrain with *Wnt1-Cre* also led to obvious forebrain phenotypes by E14.5 where the anterior cranium is slightly expanded in *Wnt1-Cre; Kif3a*^*flox/flox*^ mutants (Fig 7A and 7B; 109% of control, p = 0.029). Histological analysis revealed significant ventriculomegaly and a widened floor of the third ventricle (Fig 7C and 7D). Neurogenesis did not appear to be disrupted as the pattern of TuJI immunoreactivity appeared similar between mutant and control (Fig 7E and 7F). These abnormalities continued through development and E18.5 mutants had very dysmorphic heads (Fig 7G and 7H) and expanded forebrain (112% of control, p = 0.0229) and midbrain tissue (Fig 7I and 7J). Histological analysis confirmed the mutants had significant ventriculomegaly, reduced hippocampal development and reduced production of mature neurons as evident by a reduced cortical plate (Fig 7K and 7L).

We observed similar, but more dramatic, deficits in *Ap2-Cre; Kif3a*^*flox/flox*^ mutants where the Cre recombination pattern extends beyond the neural crest and also includes the surface ectoderm (Fig 1R–1T). In these mutants, the anterior expansion was much more noticeable at E14.5 than the *Wnt1-Cre* mediated recombination (Fig 7M and 7N; 184% of control, p = 3.48E-7). Histology and TuJI immunohistochemistry revealed that neurogenesis was profoundly disrupted as there appeared to be a significant expansion of the ventricular zone (indicating hyper-proliferation) in the *Ap2-Cre; Kif3a*^*flox/flox*^ mutant telencephalon as compared to controls (Fig 7O–7R). These abnormal phenotypes are even more dramatic at E18.5 (Fig 7S–7X). In addition to the severe shortening of the snout, *Ap2-Cre; Kif3a*^*flox/flox*^ mutants lacked olfactory bulbs and had massively expanded forebrains (Fig 7U and 7V; 190% of control, p = 1.45E-4). Histological analysis confirmed ventriculomegaly and disorganized cortical tissues consistent with abnormal patterns of neurogenesis (Fig 7W and 7X).

### *Ift88* conditional ablations reveal different spatiotemporal requirements than either *Ttc21b* or *Kif3a*

**Fig 8:**
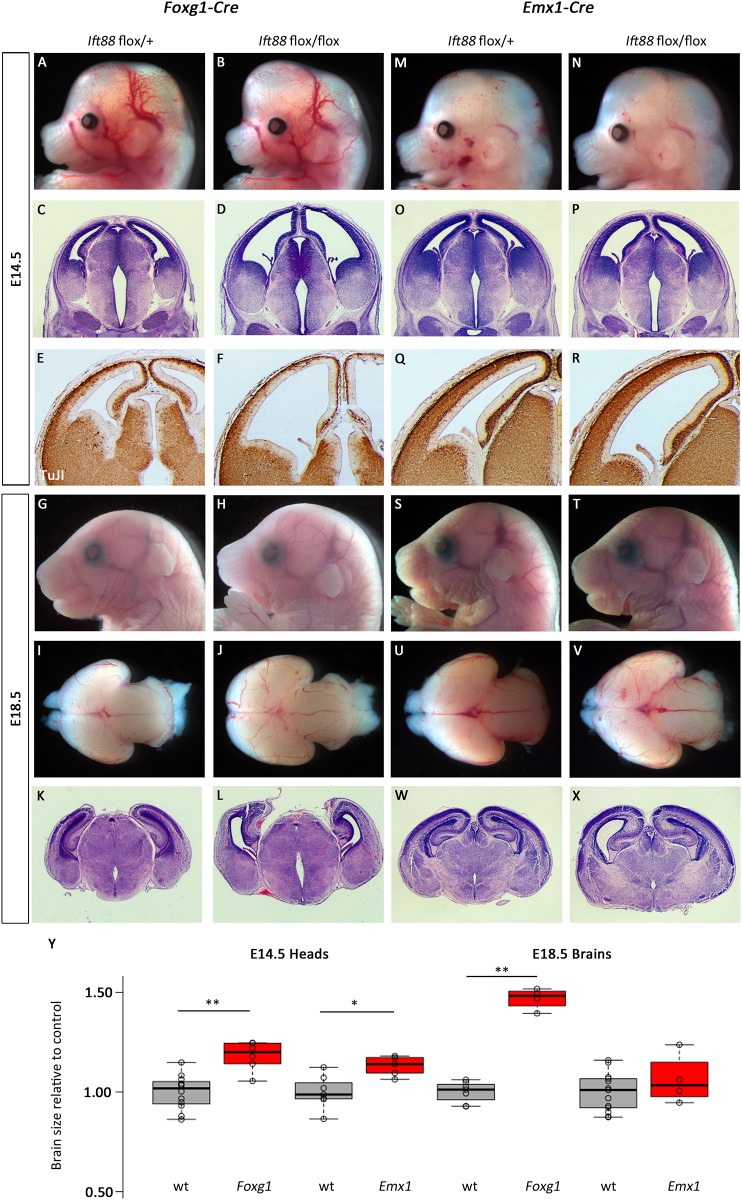
Deletion of *Ift88* in early stages of forebrain development leads to increased brain size. (A-L) *Foxg1-Cre* was used to delete a conditional allele of *Ift88* and the forebrain was enlarged at E14.5 (B) and E18.5 (H,J,). Cortical architecture is disrupted at all stages examined (D,L) and fewer differentiated neurons are seen at E14.5 (F). (M-R) Similar, but less severe, phenotypes are seen with *Emx1-Cre* ablation at E14.5. (S-X) E18.5 mutants appear phenotypically normal. All paired images are at the same magnification. (Y) Quantification for brain sizes. Center lines show the medians; box limits indicate the 25th and 75th percentiles as determined by R software; whiskers extend 1.5 times the interquartile range from the 25th and 75th percentiles, outliers are represented by dots; data points are plotted as open circles. n = 12, 6, 7, 5, 7, 4, 13, 4, respectively. (*:p = 0.011, **:p<0.005).

We next tested the hypothesis that the differences in phenotypes between the *Ttc21b* and *Kif3a* ablations are simply a result of the genes different transport functions within the cilium (retrograde and anterograde transport). We used a conditional allele of *Ift88* to genetically remove this member of the IFT-B complex which has been previously shown to be required for proper cilia form and function through anterograde transport. Similar to our *Kif3a* results, early ablation (E9.5) of *Ift88* in the mouse forebrain with *Foxg1-Cre* affects forebrain development. *Foxg1-Cre; Ift88*^*flox/flox*^ mutant embryos exhibited a dramatic increase in anterior forebrain tissue (Fig 8A and 8B; 118% of control, p = 0.0004) which was again shown to be the result of ventriculomegaly upon histological analysis (Fig 8C and 8D). However, the reduction in differentiated neurons in the *Foxg1-Cre; Ift88*^*flox/flox*^ mutants at E14.5 was more severe than that seen in *Foxg1-Cre; Kif3a*^*flox/flox*^ embryos (Fig 8E and 8F). These anterior phenotypes were still notable at E18.5 (Fig 8G and 8H). Microdissection and histological analysis of the brain did reveal a lack of olfactory bulbs and increased forebrain (Fig 8I and 8J; 146% of control, p = 2.08E-7) as well as continued ventriculomegaly affecting the telencephalic and third ventricles and abnormal tissue architecture within the cortical plate (Fig 8K and 8L).

Consistent with our other results, genetic ablation of *Ift88* only approximately one day later with *Emx1-Cre* resulted in much less dramatic effects. *Emx1-Cre; Ift88*^*flox/flox*^ mutant embryos had more subtle gross morphological defects (Fig 8M and 8N; 113% of control, p = 0.01) and mild ventriculomegaly (Fig 8O and 8P). Neurogenesis and differentiation was largely normal as indicated by the patterns of TuJI immunoreactivity (Fig 8Q and 8R). E18.5 *Emx1-Cre; Ift88*^*flox/flox*^ mutant embryos were largely indistinguishable from littermates at E18.5 (Fig 8S and 8T). More detailed analysis of the brain showed only a slight increase in forebrain size (Fig 8U and 8V; 106% of control, p = 0.31) and mild ventriculomegaly with grossly normal cortical tissue architecture. (Fig 8W and 8X).

**Fig 9:**
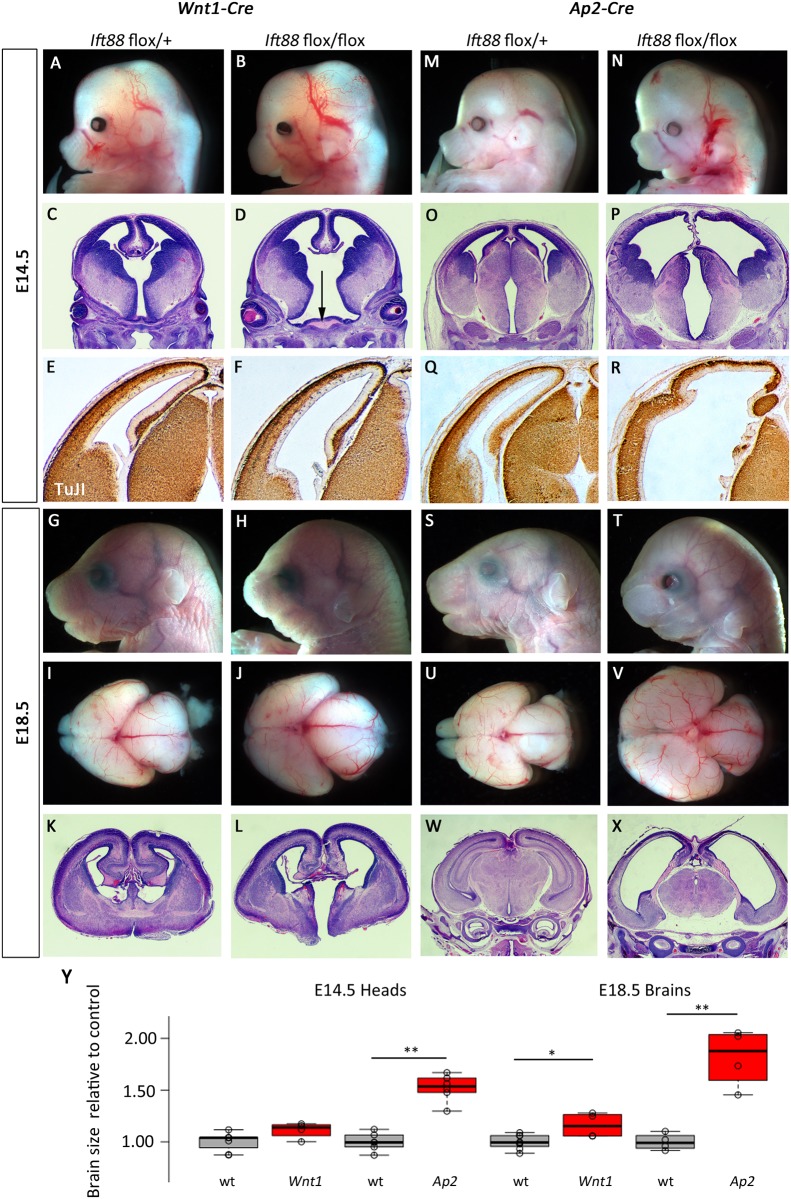
Deletion of *Ift88* from neural crest and surface causes cortical malformation. (A-L) *Wnt1-Cre* mediated deletion of *Ift88* causes morphological (A,B,G,H,I,J), and histological (C,D,K,L) phenotypes in the forebrain at E14.5 (A-F) and E18.5 (G-L). The third ventricle is enlarged at E14.5 (arrow indicates widened base of ventricle in D) and cleft at E18.5 (L). (M-X) *Ap2-Cre; Ift88*^*flox/flox*^ embryos at E14.5 and E18.5 have a profoundly enlarged forebrain (N, V) with disrupted cortical architecture (P,R,X). All paired images are at the same magnification. (Y) Quantification for brain sizes. Center lines show the medians; box limits indicate the 25th and 75th percentiles as determined by R software; whiskers extend 1.5 times the interquartile range from the 25th and 75th percentiles, outliers are represented by dots; data points are plotted as open circles. n = 7, 4, 6, 6, 8, 4, 4, 4, respectively. (*:p = 0.013, **:p<0.002)

Genetic ablation of *Ift88* with neural crest Cre transgenes again had very dramatic effects on neural development. *Wnt1-Cre; Ift88*^*flox/flox*^ mutant embryos were readily recognizable upon dissection (Fig 9A and 9B) had a widening of the ventral midline (Fig 9C and 9D) but we saw no effects on patterns of neural differentiation (Fig 9E and 9F). *Wnt1-Cre; Ift88*^*flox/flox*^ mutant embryos at E18.5 had clearly dysmorphic heads (Fig 9G and 9H) and expanded forebrains with severely hypoplastic olfactory bulbs (Fig 9I and 9J; 16% increase in forebrain size,p = 0.013). Histology showed relatively normal patterns of cortical neurogenesis but a dramatic cleft in the ventral brain (Fig 9K and 9L).

Consistent with the expanded domain of Cre activity in the surface ectoderm, *Ap2-Cre; Ift88*^*flox/flox*^ mutant embryos had more dramatic phenotypes at all stages examined. At E14.5, mutants had a dramatically enlarged forebrain (Fig 9M and 9N; 152% of control, p = 1.99E-5). Histological and immunohistochemical analyses indicated ventriculomegaly throughout the first three ventricles and disrupted patterns of neurogenesis in the cortical plate (Fig 9O–9R). E18.5 *Ap2-Cre; Ift88*^*flox/flox*^ mutant embryos were easily identified from littermates (Fig 9S and 9T) with an 81% increase in forebrain size (p = 0.001) with significant ventriculomegaly and loss of olfactory bulbs (Fig 9U and 9V) and the most significant loss of cortical tissue of any genotype examined in this study (Fig 9W and 9X).

Taken together, these results show that discrete ablations of three proteins with well-established roles in primary cilia form and function result in very different anterior neural phenotypes. Even with two genes thought to largely function similarly in anterograde transport, notable differences were seen between *Kif3a* and *Ift88* mutants. The details of these phenotypes are summarized in Table 1.

**Table 1:** 

		E14.5					E18.5					
		Forebrain size	Lateral Ventricles	Cortical Morphology	Neuronal Differentiation	Basal Ganglia	Forebrain size	Lateral Ventricles	Cortical Morphology	Olfactory Bulbs	Hippocampus	Midbrain
***Ttc21b***	***Foxg1***	No Δ	No Δ	No Δ	No Δ	No Δ*	No Δ	No Δ	No Δ	No Δ	No Δ	No Δ
	***Emx1***	No Δ	No Δ	No Δ	No Δ	No Δ	No Δ	No Δ	No Δ	No Δ	No Δ	No Δ
	***Wnt1***	No Δ	No Δ	No Δ	No Δ	No Δ	No Δ	No Δ	No Δ	Hypo-plastic & laterally displaced	No Δ	Increase
	***Ap2 a***	Large increase	Increase	Dysmorphic	Reduced	Reduced	Large increase	Increased	Dysmorphic	Absent	Dysmorphic	No Δ
***Kif3a***	***Foxg1***	Small increase	Increase	No Δ	Slight reduction	Slight reduction	Large increase	Increased	No Δ	Absent	Dysmorphic	No Δ
	***Emx1***	Small increase	Small increase	No Δ	No Δ	No Δ	Small increase	No Δ	No Δ	No Δ	No Δ	No Δ
	***Wnt1***	Small increase	Increase	Slight disruption	No Δ	No Δ	Small increase	Increased	Slight disruption	Hypo-plastic & laterally displaced	Dysmorphic	Increase
	***Ap2 a***	Large increase	Large Increase	Dysmorphic	Reduced	Dysmorphic	Large increase	Increased	Dysmorphic	Absent	Dysmorphic	No Δ
***Ift88***	***Foxg1***	Small increase	Increase	No Δ	Slight reduction	Slight reduction	Large increase	No Δ	Slight disruption	Absent	Dysmorphic	No Δ
	***Emx1***	Small increase	Small increase	No Δ	No Δ	No Δ	No Δ	No Δ	No Δ	No Δ	No Δ	No Δ
	***Wnt1***	No Δ	Increase	No Δ	No Δ	No Δ	Small increase	No Δ	No Δ	Hypo-plastic	Dysmorphic	Increase
	***Ap2a***	Large increase	Large Increase	Dysmorphic	Reduced	Reduced	Large increase	Large increase	Dysmorphic	Absent	Absent?	No Δ

## Discussion

This study demonstrates unique spatiotemporal requirements in forebrain development for three genes necessary for normal form and function of the primary cilium: *Ttc21b*, *Kif3a* and *Ift88*. We used four different Cre transgenic alleles to ablate each of these genes across distinct domains within the forebrain. The wide ranging effects of the ablations revealed critical roles for these proteins with interesting differences revealed by both developmental and tissue-specific locations of Cre activity. Forebrain expansion, cortical malformations, impaired olfactory bulb development, and ventriculomegaly were among the many variably observed defects. In addition, our studies revealed that the microcephaly phenotype in homozygous *Ttc21b*^*aln/aln*^ mutants is likely due to events prior to corticogenesis and the production of defined neural progenitor cells. Taken together, our results clearly show the primary cilia are not reiteratively produced uniformly across the embryos transducing canonical pathways in identical ways. Rather, these seem to be more strategically employed to regulate signaling during organogenesis.

### Comparison to previous neural phenotypes

Our work complements and extends prior studies on the role of these gene in cortical development. First, the mechanism of the neural phenotypes revealed by loss of *Ttc21b* is not fully understood. Our study demonstrated that neither ablation of *Ttc21b* from the forebrain at E9.5 or E10.5 phenocopied the microcephaly observed in *Ttc21b*^*aln/aln*^ homozygous mutants. Interestingly, ablation of *Ttc21b* in the brain and surrounding domains (NCCs and surface ectoderm) as early as E8.5 with the *Ap2-Cre* served only to increase brain size (Fig 5). The mosaicism of the *EIIa-Cre* germline ablation provided further evidence that loss of *Ttc21b* in the brain is not responsible for the microcephaly phenotype (Fig 3). Together, these findings suggest a role for *Ttc21b* in brain patterning and growth prior to organogenesis stages. Supporting this hypothesis are the findings of early widespread expression of *Ttc21b*, suggesting the critical role might be during the neural plate stage of development.

A previous study showed ablation of *Kif3a* using a GFAP-Cre resulted in aberrant Gli activity and cortical overgrowth [33], but the specific transgene used has been shown to be expressed throughout the early embryo which precludes any conclusions about specific lineages and/or spatiotemporal requirements for *Kif3a* beyond gastrulation [34]. A separate experiment using a GFAP-Cre transgene that initiates recombination around E13.5 [35] did not result in any appreciable differences in forebrain size or morphology [36]. The experiments we present are based on Cre recombinase activity at stages between the GFAP-Cre domains of these earlier studies. Also, similar to our work, an *Emx1-Cre; Ift88* ablation did not result in significant changes in brain size [37] and homozygous mice for a hypomorphic allele of *Ift88 (Ift88*^*cbs/cbs*^*)* have strong similarities to *Ttc21b*^*aln/aln*^ mutants [38]. The other embryonic requirements for *Ift88* presented herein have not been demonstrated. Interestingly, *Arl13b* is required for proper axoneme structure and Shh signal transduction within the primary cilium [39] but loss of *Arl13b* within the cortical epithelium had dramatic effects on the polarized radial progenitor scaffold unlike the phenotypes we observe [40]. In a similar experimental paradigm to what we show here, *Arl13b* deletion after radial progenitors were established had little effect on cortical morphology [40]. Together, these findings also suggest a role for cortical patterning by *Ttc21b*, *Kif3a*, and *Ift88* outside the cortex itself.

### Phenotypes caused by loss of cilia in forebrain tissues suggest involvement of Hh and Wnt signaling activity

Patterning of the neural plate is a crucial early step in proper brain development. The embryonic anterior neural plate (ANP) generates the forebrain and its exposure to, and protection from, various patterning molecules are decisive in this process [41, 42]. One critical signaling pathway which must be regulated in the ANP is canonical Wnt signaling. The Wnt pathway is active throughout the posterior embryo and a rostral expansion of Wnt results in a caudalizing of the anterior embryo including the head and brain [43]. Primary cilia have been previously implicated in modulating Wnt signaling [44]. In *Ttc21b*^*aln/aln*^ fibroblasts, specifically, increased activation of the Wnt pathway has been shown to occur in the presence of ligand as compared to controls [9]. Together with this previous data, our study suggests that excessive Wnt activity in the early *Ttc21b*^*aln/aln*^ embryo may be a major contributor to the microcephaly.

The role of primary cilia in transducing another crucial developmental pathway, Shh signaling, has been well established [1, 45]. The Shh pathway has been shown to be upregulated in *aln* embryos later than the neural pate stage, but has not yet been explored at this stage [6]. Shh is active at the neural plate stage, but remains restricted to the axial midline [46]. Interestingly, this domain contains an important signaling center for ANP development, the axial mesendoderm (AME). The AME is a source of multiple signals which serve to induce the anterior forebrain and protect it from caudalizing influences, including Wnt signaling [41, 46]. Shh signaling has been shown to affect AME signals, offering another potential method by which the *aln* mutation may be disrupting forebrain development [47, 48]. Increased Shh signaling in the cortex does result in progenitor expansion and cortical folding in a manner somewhat similar to the *Ap2-Cre;Ttc21b*^*flox/aln*^ phenotype [49].

With multiple AME signals functioning as Wnt inhibitors, an indirect regulation of the Wnt pathway via a primary disruption in Shh signaling is a possibility. Together, the study of both Shh and Wnt pathways in early developing *Ttc21b*^*aln/aln*^ mutants represent interesting future areas of research into novel mechanisms of microcephaly.

Similar to *Ttc21b*, both *Kif3a* and *Ift88* are known to be critical for proper *Shh* signaling. Unlike the upregulation of the *Shh* pathway seen in *Ttc21b*^*aln/aln*^ mutants, both *Kif3a* and *Ift88* mutants have been observed to have reduced Shh pathway activity [3],[1]. However, in some specific tissues, loss of *Kif3a* and *Ift88* can cause domain specific upregulation of the Shh pathway, which complicates any extrapolation of the data into other potential interpretations [15, 50]. Indeed, our work would further suggest tissue-specific responses of the Shh pathway to loss of ciliary proteins. In the *Kif3a* and *Ift88* ablations using *Ap2-Cre* and *Wnt1-Cre*, we observed an expansion of the ventral midline and third ventricle of the brain, consistent with an increase in Shh signaling. This midline hyperplasia is not observed in the *Ap2-Cre;Ttc21b*^*flox/aln*^ and *Wnt1-Cre; Ttc21b*^*flox/aln*^ embryos, despite the generalized association between *Ttc21b* and increased Shh pathway activity.

Additionally, we observed a mild dorsalization of *Kif3a* and *Ift88* using *Foxg1-Cre* along with the loss of the ventral basal ganglia, indicative of disrupted *Shh* pathway in the forebrain. In the dorsal forebrain the Shh pathway is controlled primarily by Gli3 expression, rather than by the Shh ligand itself, and low Shh activity is consistent with dorsal fates. One potential explanation for the increased dorsal telencephalon in these *Foxg1-Cre; Kif3a/Ift88* mutants is an increase in the domain of Gli3 repressor activity. Canonically, Gli3 functions as a repressor of downstream Shh pathway genes in the absence of Shh [45]. All three ciliary proteins described in our study have been shown to affect Gli3 processing [8, 33, 51]. Therefore, any disruptions of the Shh pathway may be a result of improper transduction in the presence of Shh signal, failure to properly process Gli3 in regions of low Shh activity, or likely a combination of both. Shh and Gli3 play a number of important roles in the development of the forebrain and central to these processes lies the primary cilium. We have shown the wide-ranging outcomes of these signals which result from differentially impairing cilia based on time, location, and composition.

This study also provides insight into differences between anterograde (*Kif3a*, *Ift88*) and retrograde (*Ttc21b*) trafficking within the cilium. Ablating *Ttc21b* has an almost universally less severe impact on brain development than the ablations of the anterograde transport genes. This is not surprising in that cilia, although shortened and impaired, are still produced in *Ttc21b* mutants, while cilia are not produced, or are severely disrupted in either anterograde mutation [8, 36, 52, 53]. Differences in head and brain size are noticed between the *Kif3a* and *Ift88* in the various genetic ablations as are forebrain structural defects. In each case however, *Kif3a* ablation is observed to have a more severe phenotype than loss of *Ift88*. This too may be explained by differential effects on ciliary formation. In *Kif3a* perturbations, the basal body of the cilium attaches to the cell surface but no microtubules are projected while severely truncated microtubules project from a basal body no further than the transition zone in *Ift88* mutants [36, 52, 53]. The distal tip of the cilium is known to be crucial for the Shh pathway regulatory role of primary cilia and its absence in these anterograde mutations may explain why both suffer similar Shh signaling disruptions [54]. The ciliary membrane is increasingly being shown to play a unique and important signaling role. The increased area of potential ciliary membrane, which results from the slight projection of *Ift88* conditional mutants, may preserve an important function missing in *Kif3a* mutants, explaining the increased severity of our *Kif3a* ablations. Future studies on the mechanistic roots of the phenotypes we show here are likely to further enhance our understanding of the role for primary cilia proteins in forebrain development.

Severe defects in both the morphology and neuronal differentiation of these mutants display just how crucial each protein is for proper forebrain development and the importance of the domain and timing of loss. It is known, but underappreciated, that ciliary genes are not all ubiquitously expressed (e.g., [6]). The differences we see here between different genetic ablations highlight the idea that primary cilia may be populated by different proteins in different tissues. This would further demonstrate the primary cilium is not a static organelle repeatedly employed by the embryo to relay information in a standard way. By comparing the different ablations, we can begin to parse out the temporal or regional effects that cause the cortical malformation. By comparing the recombination of *Ap2-Cre* to *Wnt1-Cre*, we can eliminate the NCCs as a responsible domain as these phenotypes are not seen in *Wnt1-Cre*- ablations. This leaves *Ap2-Cre* activity within the ectoderm (both surface and neuroectoderm) as the responsible domain(s) for the phenotype. Two differences remain between the *Ap2a-Cre* and *Foxg1-Cre* ablations: loss within the surface ectoderm and an earlier ablation (by about a day) within the prospective forebrain tissue. We attempted to address the surface ectoderm specifically in our experiments by using the Cre-ectoderm driver, as used in Schock et al., (accompanying manuscript). However, the recombination pattern was quite variable in our hands precluding an effective experiment to address this hypothesis. An alternative route to this answer is an ablation specific to the prospective forebrain at E8.5. Taken together, our experiments clearly show that ciliary protein signaling is crucial for forebrain development in multiple specific spatiotemporal domains of the early embryo. We further demonstrate that ablating similarly acting genes within the cilia can result in strikingly disparate phenotypes from each other and from different ablation time points, suggesting that parallels can only be loosely drawn from experiments in other developmental settings.

We are largely interpreting our results based on a model in which the predominant function of these genes is within the primary cilium. An alternative, or perhaps complementary, model would allow for a role for these proteins outside the primary cilium. Although these ciliary roles are the best established for *Ttc21b*, *Ift88* and *Kif3a* in embryonic development, the literature clearly indicates we should consider non-ciliary roles as well. The immunocytochemistry analysis of TTC21B localization does clearly show protein is not restricted to tubulin-positive primary cilia [8]. The heterotrimeric kinesin motor is known to play a role in transport along axons, opsin transport in photoreceptors, transporting virus within the cell, and transport of molecules required for cell-cell adhesion (among other functions, see [14, 55] for a full discussion). This represents an area of future investigation for our labs as well as many others. There are multiple mechanisms by which these non-ciliary functions could alter forebrain development in ways such as we demonstrate here.
